# The Impact of Haze Pollution on Regional Eco-Economic Treatment Efficiency in China: An Environmental Regulation Perspective

**DOI:** 10.3390/ijerph16214059

**Published:** 2019-10-23

**Authors:** Jian Hou, Yifang An, Hongfeng Song, Jiancheng Chen

**Affiliations:** School of Economics and Management, Beijing Forestry University, Beijing 100083, China; houjian1128@bjfu.edu.cn (J.H.); anyifang9535@163.com (Y.A.); chenjc1963@163.com (J.C.)

**Keywords:** haze pollution, eco-economic treatment efficiency, forcing mechanism, environmental regulation, dynamic threshold model, China

## Abstract

“The Gray Great Wall” formed by haze pollution is an increasingly serious issue in China, and the resulting air pollution has brought severe challenges to human health, the socio-economy and the world ecosystem. Based on the facts above, this paper uses China’s province-level panel data from 2009 to 2016, systematically measures the heterogeneous structure of regional ecological economic (eco-economic) treatment efficiency through a Super Slacks-Based Measure (SBM) model and dynamic threshold models, and analyzes the forcing mechanism of haze pollution pressure on regional eco-economic treatment efficiency from an environmental regulation perspective. Results indicated that China’s eco-economic treatment has been vigorously promoted, which is significantly conducive to green growth upgrading. However, the process has a large developmental scope due to regional heterogeneity. Interestingly, the forcing impact of haze pollution on regional eco-economic treatment efficiency is limited by the “critical mass” of environmental regulations: a weak degree of regulation will facilitate an increase in regional eco-economic treatment efficiency through the forcing effect of haze pollution pressure. Once environmental regulation reaches a critical level, a stronger degree of regulation will suppress the forcing effect of haze pollution in turn and it will decrease the regional eco-economic treatment efficiency. This paper endeavors to clarify the differences, suitability and dependency in the process of ecological transformation for Chinese local governments in different regions and provide policy references for a regional ecological transformation matching system.

## 1. Introduction

Haze pollution, which mainly comprises particulate matter ≤10 µm (PM10) and particulate matter ≤2.5 µm (PM2.5) in aerodynamic diameter [1,2], is a particularly serious issue. Haze is a type of atmospheric phenomenon that is formed by the accumulation of dust and smoke particles in relatively dry air, and the resulting atmospheric pollution is increasingly being highlighted in recent years [3]; it also poses a severe challenge to human health, the socio-economy and the ecosystem globally. In China, heavy haze pollution causes 1.2–1.6 million premature deaths each year [4,5]. By the end of 2017, air quality monitoring showed that only 29.3% of 338 cities in China met the standards—that is, the overall situation remained grim. However, due to the long-term impact of energy consumption and sustained economic growth, haze pollution in China will continue for a certain period of time. Facing global green and low-carbon initiative competition, as well as the severe situation of insufficient domestic environmental carrying capacity, the performance of the Chinese government in terms of haze pollution control has become the focus of the international community.

Given that “The Gray Great Wall”, formed by current haze pollution, is increasingly being highlighted, the optimization of ecological economic (eco-economic) treatment is the key link in regional green transformation [6]. In September 2013, the State Council of China launched the National Air Pollution Control Action Plan (NAPCAP) to clarify the goal of haze pollution control for the first time. Then, the enactment of a series of laws and policies, including the “Air Pollution Prevention Law”, the “Three-Year Action Plan for Winning the Blue Sky Defense War” and the “Measures for the Administration of Special Funds for Air Pollution Prevention and Control” further highlighted the great importance that the Chinese government attached to the issue of haze pollution. In addition, local governments have also made important innovations in regulation policies related to haze pollution, introduced a rapid policy update mode for eco-economic treatment, and successively implemented the coordinated ecological treatment of haze.

Wang et al. [7] noted that the environmental pollution caused by modern social and economic development has become increasingly serious, and regional green development relies to a large extent on local governments’ series of reasonable environmental policies. The reason is that environmental pollution behavior is an externality in the process of production and consumption and requires supplementary restrictions in the form of environmental regulation [6]. However, Sinn [8] initiated the “Green Paradox”, which caused scholars to question the necessity and effectiveness of environmental regulation. Meanwhile, there are great differences in resource endowment, population size, economic development, energy and industrial structure among provinces in China, and there are many factors driving the optimization of eco-economic treatment. Under the constraints of environmental regulation, the effect of these factors on the relationship between haze pollution and regional eco-economic treatment may be changed both in direction and intensity. Therefore, considering the different degrees of environmental regulation in China, what are the forcing mechanism and endogenous differences in haze pollution as they relate to regional eco-economic treatment efficiency? Will haze pollution pressure promote an increase in regional eco-economic treatment efficiency effectively in turn? How do we coordinate the contradiction between environmental protection and economic growth, thus guiding regional green development through the eco-economic treatment of haze pollution under effective environmental regulation? Due to the global low-carbon competition and development push, it is of theoretical value and practical significance to scientifically assess the regional eco-economic treatment efficiency and explore the driving factors, regulatory paths and policy designs for promoting the reduction of haze pollution.

We attempt to analyze the forcing linkages between haze pollution, environmental regulation and regional eco-economic treatment efficiency by considering the perspective of the “threshold effect”. First, based on China’s province-level panel data from 2009 to 2016, we systematically measure the heterogeneous structure of regional eco-economic treatment efficiency by using a Super- Super Slacks-Based Measure (SBM) model, which considers the undesirable output. Second, as the prominent heterogeneity among different provinces in China, we assume that a nonlinear relationship exists between haze pollution and regional eco-economic treatment efficiency. Under the perspective of environmental regulation, the use of a dynamic threshold model sheds light on how different degrees of regulation affect the relationship between haze pollution and regional eco-economic treatment efficiency, and whether threshold values exist in this relationship. The results provide policy-making references for a regional ecological transformation matching system.

## 2. Literature Review

With the acceleration of industrialization and urbanization, China’s haze pollution is an increasingly serious issue, and the concentration of fine particulate matter is generally high, which has posed great challenges to environmental treatment. Zhang and Li [9] found that haze pollution has a significantly negative impact on economic development. On average, in 2015, when other conditions remain unchanged, a 5 μg/m^3^ increase in the concentration of PM2.5 may result in a reduction in per capita Gross Domestic Product (GDP) of approximately 2500 RMB. In addition, sustainable economic growth helps to decrease PM2.5 concentrations, which in turn contributes to economic development. However, Yuan and Huang [3] studied the effects of urban morphology on haze pollution in China based on a spatial regression analysis of PM2.5 remote sensing data. The results showed that the impact of population density and centering on air quality depends on the size of the population, and the transmission among regions is an important source of haze pollution; thus, a joint management strategy for regional haze pollution should be developed. At the micro level, Hao et al. [10] and Li et al. [11] further explored the market’s response to haze-related regulatory cost expectations, emphasizing the impact of haze on corporate control and decision making, and they concluded that haze attracts the attention of investors through their direct physical and psychological experience, related news and government regulations, and then influences the stock market. However, since the market is unsuccessful in determining the external economic behavior of pollution emissions, the effective treatment of haze pollution relies to some extent on the supplementary restrictions of relevant policies. Shen and Wang [12] discussed the regulatory mechanism of corporate pollution behavior in China and established two types of government supervision mechanisms through an evolutionary game. They concluded that “long-term supervision” is better than “centralized supervision”. Furthermore, from the perspective of local governments, Yang et al. [13] proposed that they can use environmental regulations to achieve economic goals and adopt a more comprehensive and fair policy design to address China’s environmental equity issues. However, there are also scholars such as Ma et al. [14] who utilized the theoretical framework between regional innovation, environmental regulation and carbon pressure level, finding that China’s carbon pressure level has changed from a surplus to overload, while environmental regulation has a threshold effect on the regional environmental pollution, and the changes of carbon pressure levels between different thresholds will have different effects on it. Therefore, differentiated environmental regulation policies must be implemented to realize the low carbon transformation goal.

The benign eco-economic treatment model is the most effective approach for solving the current issue of haze pollution. However, right now, there is no unified definition of effective eco-economic treatment. One viewpoint advocates the government-leading treatment approach, while the other advocates a market-based treatment approach. Nevertheless, in light of the actual situation, neither of these two approaches can fundamentally solve the problems in the current Chinese ecological environment and, according to the characteristics of ecological environment treatment, the multi-subject participation treatment model has become an inevitable choice. First, the pursuit for an environmentally-friendly and sustainable development model and industrial green transformation is the essential focus for enterprises to participate in industrial upgrading and social responsibility undertaking. Further, Hillman et al. [15] stated that as a community-driven approach, social businesses are located in the third sector of the economy, often where there is market or government failure in providing social welfare, and are increasingly becoming a key driver of social progress. Second, Yuan and Guo [16] argued that the ecological treatment should be further improved by perfecting the governments’ coordinated ecological treatment system, eliminating the barriers of public ecological treatment participation, adhering to the concept of sustainable ecological development, and coordinating ecological and economic support. However, Zhang and Li [9] believe that due to the heterogeneity of governments, it is impossible for different governments to form a stable cooperation model spontaneously. In view of this, superior governments should impose administrative punishment on uncooperative governments and promote the formation and stability of the cotreatment model. However, Guttman et al. [17] argued that the ability of governments to solve environmental problems is limited, thus leading to the rise of various “nonstate parties” to supply complementary efforts or provide alternative mechanisms for tackling environmental problems.

In terms of the topic of this paper, relevant studies on the relationship between haze pollution and eco-economic treatment can be roughly divided into two perspectives according to mechanism: one is the role of government eco-economic treatment in reducing haze pollution and the other is the forcing mechanism of haze pollution on the establishment of environmental protection mechanisms at all levels of government.

From the former perspective, some scholars first measured the impact of environmental protection policies on environmental pollution from the overall government level, and noted that environmental protection measures indeed alleviate a certain degree of pollution, but the degree is not large, so further efforts of the government are still needed [18,19,20,21,22,23,24]. Some scholars also studied the relationship between the industrial structure and energy use and haze, demonstrating that the transformation of the industrial structure and changing energy usage types are important measures to control China’s heavy haze pollution [25]. When focusing on specific policies, Tang et al. [26], Huang et al. [27] and Chen et al. [28] studied the impact of environmental taxes and subsidies on enterprises, and noted that it is an effective environmental pollution control policy to impose pollution taxes on those polluting factories and utilize the taxes to subsidize high-productivity and innovation-driven enterprises. Meanwhile, Auffhammer and Kellogg [29], Zheng et al. [30], Willis et al. [31] and Vagnoni and Moradi [32] studied the effect of local governments’ environmental protection policies on environmental treatment and found that the establishment of local environmental protection mechanisms has a positive impact on the decrease in haze pollution.

From the second perspective, however, research on the forcing mechanism of haze pollution pressure on the ecological treatment has been neglected for a long time, which is relative to the first perspective. Nevertheless, some scholars have begun to put forward the theoretical concept of the “forcing effect on emission reduction”. In other words, environmental regulation will promote the transfer and structure upgrading of polluting industries as long as pollution reaches the threshold value, effectively forcing the industrial structure toward the direction of low carbonization [33]. The concept of a “forcing effect on emission reduction” represents a novel perspective on the construction of the government function model, thus introducing new ideas for China to more effectively achieve its multi-subject ecological treatment goal.

Based on the previous studies summarized above, this paper fills in the research gaps as follows. First, although the relevant research on green transformation has formed a certain foundation, most research focuses on studies about positive pollution improvement, generally ignoring its forcing path to environmental treatment. In particular, the research on heterogeneous impacts between regions in China is very scarce, and relevant data, model tools and empirical analysis are even minimal. In contrast, based on the definition of the haze pollution space–time path and eco-economic treatment characteristics, we mainly explore the forcing effect of haze pollution pressure on regional eco-economic treatment efficiency, and develop a comparative analysis about treatment optimization plans in different regions.

Second, existing studies on China’s eco-economic treatment effectiveness are usually limited to the calculation of government or enterprise green development efficiency in a broad sense, while there is little among the literature on structural optimization differences in regional ecological treatment. We fill this gap by adopting the newly developed Super-SBM model, considering undesirable output to measure the structural differences and characteristics of regional eco-economic treatment, which can overcome the structural distinction matter when the treatment in different regions is effective simultaneously [34], and make this study more scientific and rational.

Third, under the circumstance of China’s regional environmental policy and resource distribution heterogeneity, the green ecological civilization development systems in different regions have their own characteristics [35], and different degrees of environmental regulation affect green transformation in different ways—that is, any type of regulatory path should match the heterogeneous threshold of regional eco-economic treatment well. However, most of the existing literature tends to focus on the statically linear relationship between haze pollution and eco-economic treatment in isolation, and almost never considers the heterogeneous characteristics of restricted factors in a dynamic situation. In contrast, we construct a dynamic threshold model of haze pollution’s forcing mechanism in different environmental regulation threshold intervals, which effectively conquers the shortcomings of the traditional nonlinear threshold regression model that cannot reflect the dynamic change or lag effect of a sample object. In addition, heterogeneous thresholds of environmental regulation, haze pollution and eco-economic treatment efficiency in different regions are placed under the same research framework to analyze their deeply nonlinear dynamic endogenous correlations.

Overall, this paper endeavors to clarify the differences, suitability and dependency in the process of ecological transformation for Chinese local governments in different regions and provide policy references for a regional ecological transformation matching system.

## 3. Data and Methods

### 3.1. Measuring the Regional Eco-Economic Treatment Efficiency

The eco-economic treatment efficiency refers to the degree of utilization of various ecological resources aimed at achieving sustainable economic development targets. It reflects to the ability to produce more goods and services while consuming fewer natural resources and having a smaller impact on the environment [36,37]. To explore the structural differences and path characteristics of eco-economic treatment efficiency in different regions, we adopt the Super Slacks-Based Measure (SBM) model considering undesirable output, which not only better solves the problems of input–output variables’ slack and undesirable output fitting but also overcomes the structural distinction matter when the treatment in different regions is effective simultaneously [34].

We first measure the undesirable output efficiency of a non-oriented SBM of eco-economic treatment in different regions, which is:

(1) $$\begin{array}{c} \rho^{*}=min\frac{1-\frac{1}{m}\sum_{i=1}^{m}\frac{s_{i}^{-}}{x_{i_{0}}}}{1+\frac{1}{s_{1}+s_{2}}\left(\sum_{r=1}^{s_{1}}\frac{s_{r}^{g}}{y_{r_{0}}^{g}}+\sum_{r=1}^{s_{2}}\frac{s_{r}^{b}}{y_{r_{0}}^{b}}\right)}\\ \;s.t. \end{array}\;x_{0}=X\phi +s^{-}\;y_{0}^{g}=Y^{g}\phi -s^{g}\;y_{0}^{b}=Y^{b}\phi +s^{b}\;\;s^{-}\geq 0,s^{g}\geq 0,s^{b}\geq 0,\phi \geq 0$$

where Χ, $Y^{g}$ and $Y^{b}$ are the input vector, desirable output vector and undesirable output vector, respectively; $s^{-},\;s^{g}\mathrm{and}\;s^{b}$ are the input slack variable, desirable output slack variable and undesirable output slack variable, respectively; ϕ is the weight vector; $\rho^{*}$ represents the efficiency of the decision-making unit (DMU): ($x_{0},y_{0}^{g}\;\mathrm{and}\;y_{0}^{b}$), which is strictly decreasing, $0\leq \rho^{*}\leq 1$, and satisfies the following two conditions: ➀ when $\rho^{*}=1$, i.e., when $s^{-}=0$, $s^{g}=0$ and $s^{b}=0$, the DMU is valid; ➁ when $\rho^{*}<1$, which means that at least one of $s^{-},s^{g}\mathrm{or}\;s^{b}$ is not zero, the DMU is invalid, and the input–output model should be improved.

Secondly, because an undesirable output such as “three wastes” (waste gas, wastewater and industrial solid waste) pollution exists in the process of eco-economic treatment, and the eco-economic treatment in different regions could be effective at the same time. However, the calculation above does not include all DMU efficiency values and cannot measure the differentiation of decision-making units when they are effective simultaneously. In contrast, we construct the Super-SBM model, considering undesirable output to overcome this shortcoming. The set of production possibilities built by DMUs other than DMU_k_ is:

(2) $$\left\{\left(x,y\right)|x\geq \sum_{j=1,j\neq k}^{n}x_{ij}\lambda_{j},y^{g}\leq \sum_{j=1,j\neq k}^{n}y_{rj}^{g}\lambda_{j},y^{b}\geq \sum_{j=1,j\neq k}^{n}y_{rj}^{b}\lambda_{j}\right\}$$

The optimal solution of the estimated DMU_k_ in the Super-SBM model is the closest point to the leading edge in the production possibility set constructed by other DMUs, and the efficiency of DMU_k_ is recalculated according to the model after substitution:

(3) $$\begin{array}{c} \rho_{SE}=min\frac{1-\frac{1}{m}\sum_{i=1}^{m}\frac{s_{i}^{-}}{x_{i_{0}}}}{1+\frac{1}{s_{1}+s_{2}}\left(\sum_{r=1}^{s_{1}}\frac{s_{r}^{g}}{y_{r_{0}}^{g}}+\sum_{r=1}^{s_{2}}\frac{s_{r}^{b}}{y_{r_{0}}^{b}}\right)}\\ \;s.t. \end{array}\;\overset{n}{\underset{j=1,j\neq k}{\sum }}x_{ij}\lambda_{j}-s_{i}^{-}\leq x_{ik}\;\overset{n}{\underset{j=1,j\neq k}{\sum }}x_{rj}\lambda_{j}+s_{r}^{+}\geq y_{rk}\;\overset{n}{\underset{j=1,j\neq k}{\sum }}x_{bj}\lambda_{j}-s_{b}^{+}\leq y_{bk}\;s^{-}\geq 0,s^{g}\geq 0,s^{b}\geq 0,\phi \geq 0$$

Particularly, in terms of the regional eco-economic treatment efficiency (ETE) considering the undesirable output of environmental pollution, which is measured in this paper: when 0 < ETE < 1, this indicates that regional eco-economic treatment efficiency has not reached the optimal situation. In other words, the regional green transformation treatment tends to be extensive and epitaxial, thus leading to a relatively low level of eco-economic development; when ETE = 1, this indicates that regional eco-economic treatment has reached an effective state; ETE > 1 means that the desirable output of regional eco-economic treatment exceeds the capital, labor and energy inputs, which is a strongly effective state. Furthermore, it also shows that the regional treatment is intensive and connotative, and the regional economic development has realized the green transformation target [38,39].

The regional eco-economic treatment measured in this paper is based on environmental efficiency, in which the desirable output is measured by the green area and the utilization rate of solid waste, with the undesirable output being measured by the discharge amount of major industrial pollutants (industrial wastewater, exhaust emissions and solid waste discharge amount: industrial wastewater refers to wastewater and waste liquid produced in industrial process, which contains industrial materials, intermediate products, by-products and pollutants produced during industrial production; exhaust emissions is the general term for all kinds of pollutant-containing gases such as carbon dioxide, carbon disulfide and fluoride that are emitted into the air during fuel combustion and the production process; solid waste is defined as waste that is generated by businesses from an industrial or manufacturing process or waste generated from non-manufacturing activities that are managed as a separate waste stream. We collect these pollutants form the regional statistical annual reports of the Chinese government and the EPS Global Statistics Database). In addition, the three input variables include environmental protection investment, the annual operating cost of waste gas treatment facilities, and the annual treatment cost of wastewater. The data are taken from the National Bureau of Statistics of China and the National Energy Administration, which includes 30 provinces, municipalities and autonomous regions in mainland China (considering the practical situation of data shortage, we exclude the Special Administrative Regions of Tibet, Hong Kong and Macau). This paper mainly examines a series of emission reduction measures, reforms and transformations implemented by the Chinese government after the announcement of the transformation goal of a green circular economy in 2008, and given the continuity, the inertia and hysteretic nature of the process from the beginning of policy implementation to output results, we calculate the growth rate of each period, using 2009 data as the initial stage, and finally select 2009–2016 as the range for the sample.

### 3.2. Analysis of Regional Eco-Economic Treatment Efficiency

**Figure 1:**
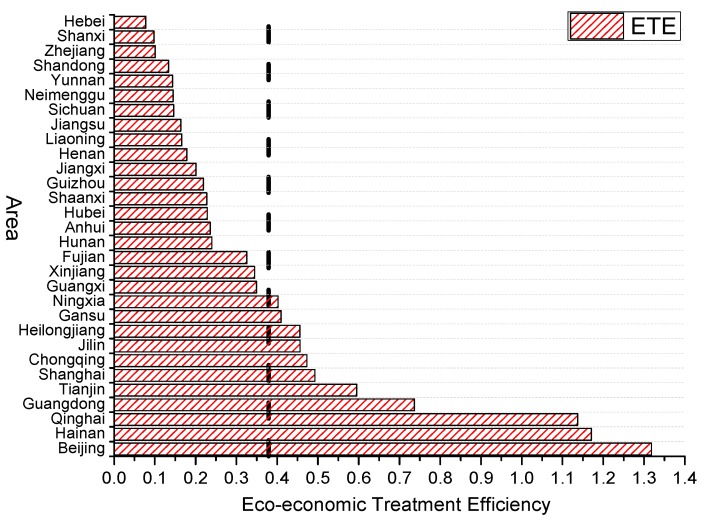
Average value of regional eco-economic treatment efficiency in China (2009–2016).

Overall, the average value of China’s eco-economic treatment efficiency (ETE) is 0.379 (the vertical line in Figure 1). This shows that the effectiveness of China’s eco-economic treatment is still low, and there is still a long way to go to achieving the goal of effective regional eco-economic treatment. In recent years, although green sustainable development has been vigorously promoted, on the one hand, China’s ecological treatment has relied heavily on the government’s investment in traditional manufacturing and infrastructure, and this continuously broad investment will greatly reduce the marginal productivity of capital; however, the serious overcapacity caused by the former extensive development mode and the problem of resource loss cannot not be solved in a short time. In addition, the economic base, human resource, industrial structure and investment of different regions are quite heterogeneous, so the eco-economic treatment failed to form a unified trend, and then led to the consequence of overall inefficiency.

Specifically, the eco-economic treatment efficiency is relatively high in Beijing, Hainan, Qinghai, Guangdong, Tianjin and Shanghai, etc. Among them, Beijing is China’s political and cultural center. Since the 2008 Olympic Games, the Chinese government has vigorously promoted the construction of the capital’s ecological civilization, constantly adjusted the industrial structure, and shut down a large number of polluting factories. The investment intensity and degree to which environmental protection has been highlighted in Beijing is far beyond that in other regions. In addition, the proportion of the secondary industry is relatively small, and the resources and environmental foundations are excellent. Under the series of measures such as promoting third-party pollution control, the efficiency of eco-economic treatment in Beijing will inevitably maintain a relatively high level. Tianjin, Shanghai, Guangdong and other developed coastal areas rely on strong economic bases and advanced technology to continuously transform the economic development mode and, at the same time, stimulate market subjects to vigorously develop cleaner production and pollution control technologies to improve the circulation rate of wastewater, waste gas and solid waste. Therefore, the eco-economic treatment efficiency of these areas is also relatively high. However, it should be noted that while actively promoting technological innovation, we must also start from the pollution source and optimize the industrial structure. The mutual cooperation of these two sides can truly effectively achieve the green growth-upgrading target. For remote areas such as Hainan and Qinghai, although they are far less developed than eastern areas in terms of talent introduction, science and technology investment, and GDP, these areas have abundant natural resources, a low degree of ecological environmental damage, and high per capita resources, so the input–output ratio of ecological treatment maintains a relatively high level. The lower rankings of Hebei, Shanxi, Shandong, Yunnan and Neimenggu have concentrated dense energy consumption and industrial pollution in China due to the limitations of the geographical environment, natural resources and their own developmental structures. A large amount of overcapacity and pollution emissions cannot be effectively solved in the short term, and the shortage of funds brought about by a weak economic foundation has made the eco-economic treatment efficiency in these areas remain at a low level [40]. Although Zhejiang Province is located in the eastern coastal areas and has a developed economy, its economic and social development is mainly attributed to many township enterprises. However, most township enterprises have lower levels of technological innovation and rely on a low-cost and low-efficiency traditional development mode, which cannot meet the treatment goal of green-drive development. In addition, the thriving economy and the innately excellent natural scenery have made the enterprises’ environmental awareness less weak and made them reluctant to change the original production mode. Therefore, although relevant measures have been taken to improve the ecological environment, the effect is not significant.

**Figure 2:**
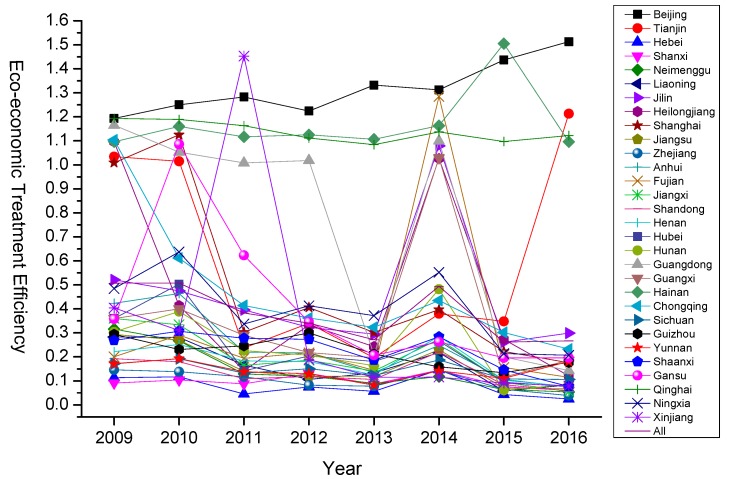
Time trends of eco-economic treatment efficiency in different regions in China (2009–2016).

Further, from a trend point of view (Figure 2), as a whole, due to the large base of environmental pollution caused by rapid economic growth, and the significant heterogeneity of regulatory bases in different regions, the current eco-economic treatment efficiency has been at a low level and cannot form a unified ecological treatment trend. In particular, in 2013, the nationwide problem of haze pollution reached its peak. Under the strong impetus of a series of government regulations and policies, the efficiency of treatment in various regions generally increased in 2014 and then fell back to a lower level. Among them, Beijing, Qinghai and Hainan have achieved a remarkable effect in ecological treatment due to their political status and innate environmental advantages respectively, and this trend will have considerable continuity in the next few years. In contrast, economic development relies mostly on traditional heavy industry in Shanxi, Hebei, Yunnan and Shandong, and although the industrial structure optimization and green cleaner technology integration has been accelerated since 2009, treatment efficiency will show some improvement in the long term. However, based on the previous energy-intensive economic base, it will remain at a low level in the near term.

### 3.3. Specifications of the Dynamic Threshold Model

Based on the theoretical framework of the regional eco-economic treatment mechanism, this paper notes that due to the significant regional heterogeneity in China, ignoring the key factors of regional environmental regulation in China will lead to deviations in the estimation of regional environmental regulation [6]. In other words, when an economic parameter reaches a certain value, it will suddenly shift another economic parameter to other forms of development. The critical mass of the root cause of this phenomenon is called the threshold value, which is also known as the problem of nonlinear “structural change”. From a methodological point of view, it is necessary to test samples on both sides of the critical mass of China’s environmental regulations. Hansen [41] first proposed the idea of the panel threshold regression model, which uses threshold variables to determine structural change points, and then forms observations to estimate the true threshold value by the bootstrapping method (under the premise that interpreting variables and threshold values are fixed, simulating a sequence of dependent variables, and each time a self-sampling sample is obtained, a simulated Lagrange Multiplier (LM) statistic can be calculated. When this process is repeated N times, we believe that the P value estimated by this “bootstrapping method” is the percentage of the number of times the LM statistic generated by the simulation is greater than the original LM statistic to the total number of simulation), thus more objectively and accurately addressing the structural changes in nonlinear problems [41]. However, this threshold method applies only to nondynamic panel models; it does not reflect the dynamic change or lag effect of sample objects, and ignores the handling of endogenous variables. Because regional eco-economic treatment is a process of transformation, to test the dynamic heterogeneity effect of haze pollution on regional eco-economic treatment, this paper adopts an improved dynamic panel threshold regression method to incorporate the lagging term of the dependent variable, which can control the continuity, inertia and model endogeneity of the ecological treatment process itself, and incorporates dynamic factors based on the dynamic panel estimation method. The threshold value is estimated based on the Hansen model, and different intervals are divided according to the threshold value. Then, the systematic Generalised Method of Moments (GMM) estimation method proposed by Blundell and Bond [42] is used to dynamically estimate the parameters between the intervals. The systematic GMM estimation combines the differential GMM with the horizontal GMM, which not only better handles the endogenous problem but also effectively reflects the early dependence and dynamic change characteristics of the eco-economic treatment.

We take the regional eco-economic treatment efficiency (ETE) as the explained variable, haze pollution (HAZ) as the core explanatory variable, environmental regulation (REG) as the threshold variable, and incorporate the lagging term of ETE and a series of control variables such as energy intensity (ENE), urbanization (URB), industrialization (IND), foreign direct investment (FDI) and innovation effect (INO) to investigate the forcing effect of haze pollution on eco-economic treatment under different thresholds of environmental regulation. We set the dynamic panel threshold model (taking a single-threshold model as an example) as follows:

(4) $$ETE_{it}=\theta +\alpha_{1}ETE_{it-1}+\alpha_{2}ETE_{it-2}+\alpha_{3}URB_{it}+\alpha_{4}ENE_{it}+\alpha_{5}FDI_{it}+\alpha_{6}IND_{it}+\alpha_{7}INO_{it}\;+\beta_{1}HAZ_{it}I\left(REG_{it}\leq \gamma \right)+\beta_{2}HAZ_{it}I\left(REG_{it}>\gamma \right)+\mu_{i}+\nu_{t}+\varepsilon_{it}$$

where I(⋅) is the indicator function; γ is the threshold value; $\mu_{i}$ is a specific effect of the individual; $\nu_{t}$ is a specific effect of time; and $\varepsilon_{it}$ is a random disturbance. It should be noted that, by stepwise calculation, it was determined that the second-order lag equation is significant and the fitting degree is the best, so we chose to further dynamically test the first-order lag.

### 3.4. Variables and Data Sources

This paper mainly measures the relationship between regional eco-economic treatment efficiency, environmental regulation and haze pollution. Among them, the regional eco-economic treatment efficiency (ETE) is measured by the Super-SBM model that incorporates undesirable output, which is the calculation result above. Regarding haze pollution, on the one hand, the last component of haze, such as nitrogen oxides, sulfur dioxide and inhalable particlea, is the main cause of increased haze pollution [43]. On the other hand, according to the China Statistical Yearbook, the PM2.5 value of major cities in China will be available only from 2012. Based on the above two points, this paper uses the annual average of PM10 to represent haze pollution (HAZ). For the measurement of environmental regulation, relevant scholars mainly use the following three approaches: the first is to measure the intensity of environmental regulation by pollutant discharge tax [44]; the second is to measure the environmental regulation intensity by the ratio of comprehensive utilization output value of “three wastes” to GDP [10]; the third is to use the Environmental Policy Strictness Index (EPS) as proxy [45,46]. Among them, the most widely used and statistically effective environmental regulation means in China is environmental protection investment. Considering the limited provincial panel data provided by the Chinese government, we use the ratio of regional environmental pollution control investment to GDP as the environmental regulation (REG).

A series of control variables were also considered in this study. First, environmental problems caused by energy consumption and pollution emissions are the primary control targets for regional eco-economic treatment efficiency. We use the ratio of energy consumption to regional GDP in each region to measure energy intensity (ENE). Second, we use the proportion of urban population to define the degree of urbanization (URB). Third, the environmental pollution problems brought about by the industrialization process make the beneficial output of environmental regulation increasingly less, which affects the effectiveness of treatment to some extent. In view of this, we use the proportion of the secondary industry to the regional GDP to represent the level of industrialization (IND). Fourth, a variable for foreign direct investment (FDI) is included, which is defined by the regional actual use of foreign direct investment. Finally, the eco-economic treatment efficiency in China is increasingly dependent on the technological innovation, especially clean production technology innovation. We use regional patent grants as a proxy for the innovation effect (INO).

Table 1 summarizes the descriptive statistics of all variables. The province-level panel data during the period 2009–2016 was obtained from the National Bureau of Statistics of China and the National Energy Administration. The nominal variables in this paper are deflated into real ones by using the GDP deflator index and fixed asset investment price deflator index, with 2009 as the base year.

## 4. Empirical Results and Discussions

### 4.1. Results of Threshold Effect Tests

This paper first tests whether there is a significant nonlinear relationship and then further determines the number of thresholds. The *F*-value and *P*-value obtained by the bootstrapping method are shown in Table 2. We find that the double threshold and triple threshold are not significant, because their *P*-values obtained by the bootstrapping method are 0.116 and 0.208, respectively. However, the single threshold is significant at the 5% level. According to Hansen’s threshold model, the relationship between haze pollution and regional eco-economic treatment efficiency has one threshold in terms of the environmental regulation.

Second, with the environmental regulation as the threshold variable, the single threshold value is 0.810, for the forcing effect of haze pollution. Moreover, the threshold estimate value is in the 95% confidence interval: 0.810[0.600, 3.010]. The specific results are shown in Table 3.

### 4.2. Estimation Results of the Dynamic Threshold Model

Based on the estimation of the significant threshold, the sample is divided into two different regimes: weakly regulated (REG ≤ 0.810) and strongly regulated (REG > 0.810). After that, this paper further estimates the slope coefficient between the regimes using the systematic GMM estimation approach proposed by Blundell and Bond [42].

Table 4 reports the forcing effect of haze pollution on regional eco-economic treatment efficiency under different degrees of environmental regulation. In the case of weak regulation (REG ≤ 0.810), haze pollution has a significantly positive impact on the regional eco-economic treatment efficiency at the 1% level. That is, the deepening of the degree of haze pollution can effectively force the improvement of regional eco-economic treatment efficiency; however, when the degree of regulation is strong (REG > 0.810), its impact mechanism has totally changed. At this time, the effect coefficient changes from positive to negative and is significant at the 1% level. The above results reflect the “critical mass” of environmental regulation in China. When the degree of regulation is relatively weak, the haze pollution pressure will force the improvement of regional eco-economic treatment efficiency. However, as the degree of regulation is further enhanced and surpasses the critical mass, the effect of haze pollution will shift to the opposite direction, which significantly reduces the efficiency level of regional eco-economic treatment.

For the other driving forces of improving regional eco-economic treatment efficiency, the urbanization level has a significantly positive impact on. URB reflects social development and advancement in science and technology in one region, as well as a gradual optimization of the local industrial structure. It is not difficult to find from the previous article that the level of eco-economic treatment efficiency in highly urbanized areas such as the eastern coastal area is generally high; ENE has a significantly negative relationship with regional eco-economic treatment efficiency, which can be explained by the fact that the higher the energy intensity, the more pollution will be aggregated, thus hindering the improvement of treatment efficiency; INO also has a significant negative impact, which is different from the general understanding. However, for most regions that are still in the early stage of innovative development in China, due to a relatively low level of technology, insufficient resource transformation has led to a heavy cost of innovation and then crowded out the treatment space to a certain extent, further decreasing the efficiency of regional eco-economic treatment; the impact of IND on the efficiency of regional eco-economic treatment is negative and very significant. Therefore, all regions should speed up the adjustment of the industrial structure and pay more attention to the balanced utilization of various kinds of limited resources to achieve the green development target; China’s FDI has also inhibited an increase in the treatment efficiency, but it is not significant. In other words, there is not enough evidence to show that the improvement in the foreign direct investment level plays a role in the decreasing regional eco-economic treatment.

## 5. Discussion

Normally, environmental regulation is necessary to improve the efficiency of regional eco-economic treatment. However, unlike previous studies, we find that the forcing mechanism of haze pollution pressure on regional treatment efficiency is more sensitive to environmental regulation due to the “scarcity” of environmental elements. Moreover, when the degree of regulation surpasses the threshold value, its impact on regional treatment efficiency will be totally the opposite. Therefore, blindly increasing the intensity of regulation cannot effectively force the improvement of regional treatment efficiency through the increase in haze pollution pressure. This paper argues that the “cost-saving” effect and “innovation offset” effect produced by different regulation intensities have become the decisive factors for adjusting the relationship between the two sides. For the Chinese context, the result of strong regulation intensity is that the crowding-out effect is greater than the offset effect. Furthermore, in the past development process, the Chinese economy has largely followed the extensive mode of heavy industry dominance with high-energy consumption, high pollution emission and low efficiency. In this condition, each region will choose to respond to environmental regulation by increasing investment in cleaner production technologies or industrial restructuring. However, no matter what method is adopted, it will inevitably increase the regional economic pressure, and then form a forcing mechanism for regional green production.

When the degree of environmental regulation is relatively weak, the standards of regulatory policies are still within the limits of enterprises. At this point, the increase in haze pollution pressure will effectively force enterprises to re-examine existing production processes, develop green energy-saving technologies and increase output rates under the influence of “innovation offset” effect, and ultimately achieve the goal of reducing enterprise cost. In addition, from the perspective of environmental regulators, a weaker regulation intensity means less supervising pressure from the government, which is beneficial to the relevant personnel to carry out their work efficiently. Based on the above two points, the target of improving regional eco-economic treatment efficiency will be achieved.

However, when the degree of environmental regulation is stronger, the haze pollution pressure will be failing in forcing enterprises to actively take relevant action to improve the treatment efficiency, because the crowding-out effect will dominate the “innovation offset effect”—that is, environmental regulation has a negative effect on the improvement in regional eco-economic treatment efficiency, which reflects the “green paradox” theory proposed by Sinn [8]. First, the excessively long-term cost produced by a strong degree of regulation will reduce the willingness of enterprises to optimize their resource management structures. For China, whose economic development is dominated by the polluting industry comprising heavy industries such as petroleum, steel, coal and chemical industries, most of these enterprises have a relatively high investment in fixed assets and a relatively high cost of technological transformation. Therefore, when the degree of regulation is gradually increased, enterprises will give priority to the strong regulatory standards through the increase in direct pollution control investment. Meanwhile, it has squeezed the space for innovation investment, which further restricts the output expansion and economic growth [47]. It can be imagined that when the regulatory standards surpass the critical mass of enterprise bearing capacity of pollution control cost, the innovation investment income will not be able to make up for the additional cost of pollution control. If these enterprises continue to meet policy standards, a series of negative externalities such as “rent seeking” will follow, resulting in a reduction in the marginal utility of environmental regulation—that is, the decrease in eco-economic treatment efficiency.

Second, we find that the law of marginal production decline in the forcing mechanism is highlighted. In the early stage, the green output of enterprises will gradually increase with the input of government ecological treatment [3]. However, as the primary goal of ecological treatment, many highly polluting enterprises rely heavily on scarce resources such as metals, coal and steel. These resources, unlike ordinary production factors, cannot release “regulatory dividends” in the short term. In view of this, with the further increase in industrial pollution control investment, particularly when the marginal cost of input is greater than the marginal green output of enterprises, although the treatment effectiveness will continue to increase within a certain period of time, the ratio of treatment effectiveness to production factor input will gradually decrease; in other words, the stronger regulation that makes the marginal cost greater than the marginal output will result in failure in the forcing effect of haze pollution pressure.

Third, excessive investment in industrial pollution control is not conducive to the decoupling of environmental pollution and economic growth, because the government’s high pollution control investment will undoubtedly form an invisible constraint on the enterprises, making them change the original production and operation mode [48]. This will make enterprises with strong profitability bear greater risks. Moreover, in the process of transformation, many enterprises have suffered losses due to market uncertainty. In other words, the increase in environmental protection investment cannot achieve an economic growth target that is not at the expense of the environment and will ultimately lead to the development of the eco-economic treatment to an inefficient path.

Overall, the effect mechanism of haze pollution on regional eco-economic treatment efficiency is different through the threshold effect of environmental regulation. Under a relatively weak degree of regulation, the increase in haze pollution pressure prompts high-polluting enterprises to adjust the production structure in time and carry out research and development of green cleaner production technology. Meanwhile, its innovative income can effectively offset the initial investment cost, thus forcing the improvement in regional treatment efficiency. In contrast, if current environmental regulation intensity is further increased beyond the regional bearing capacity, it will not only increase the pressure on policy supervision and implementers but also reduce the marginal revenue rate of pollution control and restrict the further development of enterprises with a relatively low level of technology and income. At this time, the haze pollution pressure has a restraining effect on the treatment efficiency improvement, and the ideal result of win–win situation for the economy and the environment described by the Porter hypothesis cannot be achieved.

In addition, to better achieve the upgrading of the regional eco-economic treatment efficiency, it is also necessary to exert the synergistic effect of multiple driving factors of regional treatment efficiency: Chinese governments at all levels should focus on the energy consumption characteristics of the region, establish a complete monitoring mechanism for energy intensity, and issue timely targeted treatment policies according to the energy intensity index in different periods to efficiently optimize the eco-economic efficiency. In addition, the scientific and technological resources and economic development brought about by the urbanization process are crucial to the improvement in regional eco-economic treatment efficiency. Therefore, we should pay attention to the strategic layout of regional integration construction with provincial capital taken as the core, and fully realize the extension of regional superior resources. However, for the regional industrialization level, it is necessary to speed up the shutdown of high-pollution and high energy-consuming enterprises, optimize the industrial layout, and guide the industry mode to shift from high-speed growth to high-quality growth, eventually eliminating the obstacles to improving regional eco-economic treatment efficiency.

Finally, the lag variables are significant at the 1% level, which indicates that the dynamic panel threshold model constructed in this paper is reasonable. The Hansen Test of Overid shows Prob > χ^2^ = 0.409, which does not reject the original hypothesis that the instrumental variables are reasonable at the 10% level. The Arellano-Bond (AR) (1) and AR (2) tests (Table 5) also do not reject the original hypothesis that the disturbance term $\left\{\varepsilon_{\mathrm{it}}\right\}$ has no autocorrelation; the model setting and the use of a first-order difference GMM are also more reasonable.

We also analyze the heterogeneity of environmental regulation across regions and time, which can have a better understanding of the threshold effect of environmental regulations. As shown in Table 6, the number of provinces with weaker environmental regulation (REG ≤ 0.810) showed a steady and declining trend at first, and in 2013, as the national haze pollution reached its peak, the number of weaker regulatory provinces was the lowest among all years included in this paper, and then it showed a gradually rising trend. Provinces with weaker environmental regulation (REG ≤ 0.810) account for 17.5%, while provinces with stronger environmental regulation (REG > 0.810) account for 82.5% of the total. Therefore, local governments should attach great importance to controlling the degree of regulation within a reasonable range, thus preventing the forcing mechanism of haze pollution from failing. We find that most of the provinces with stronger regulation are developed or polluted regions, such as Beijing, Tianjin, Shanxi, Hebei, Neimenggu, Zhejiang and Jiangsu. These regions have a relatively high level of industrialization, and also suffer from serious environmental pollution caused by excessive energy consumption in the past, which results in the need for more stringent regulatory measures. In recent years, more and more regions have introduced various regulations to accelerate regional green transformation. On the other hand, however, local governments should pay more attention to the regional differences in economic development and resource endowments, balance the utilization of all kinds of development factors, and fully release the bonus brought about by the appropriate degree of environmental regulation.

## 6. Conclusions and Policy Implications

Improving regional eco-economic treatment efficiency and reducing haze pollution is a task with high uncertainty and complexity. In general, the average value of China’s eco-economic treatment efficiency is 0.379. There is still a large developmental scope for China’s eco-economic treatment effectiveness, and its ecological treatment efficiency has not yet formed a unified trend, so there is still a long way to go to achieve the goal of efficient regional eco-economic treatment. The regional differentiation of China’s eco-economic treatment efficiency is significant, and not all provinces with higher efficiency levels are concentrated in the developed coastal areas of the East. The forcing mechanism of haze pollution pressure on regional eco-economic treatment efficiency is significantly constrained by environmental regulation. The weak degree of environmental regulation is conducive to the forcing effect of haze pollution on the improvement of regional eco-economic treatment efficiency. However, once the regulatory intensity breaks through the “threshold value”, the stronger degree of environmental regulation will instead suppress the forcing effect of haze pollution and reduce the efficiency of regional ecological treatment.

The conclusions of this study have some policy implications. First, in the context of global low-carbon games and low-carbon competition, improving the effectiveness of eco-economic treatment and realizing regional green growth upgrading is the key link in the process of China’s economic transformation from an extensive to an intensive development mode. Second, considering the interregional differentiation characteristics of eco-economic treatment efficiency, for the eastern coastal areas with a high overall level of treatment efficiency, they should mainly adopt the treatment method of “two-point coordinated promotion”. On the one hand, they should continue to deepen the adjustment of the industrial structure and maximize the power release of green industry. On the other hand, they should coordinate scientific and technological resources to attract and motivate R&D personnel to participate in corporate clean technology innovation. Most regions with low efficiency in eco-economic treatment should place the optimization and adjustment of the industrial structure at the core, gradually change the energy consumption structure with the high energy consumption of coal and steel as well as high pollution, and establish green corridors and clean technology industrial parks with the Chinese “Belt and Road” initiative to cultivate and develop new energy and new materials, thus finally realizing the development model of a circular economy. Third, in view of the fact that the crowding-out effect of the current strong degree of environmental regulation is greater than the innovation offset effect, in areas with strong regulation intensity, in addition to establishing an early warning mechanism for haze pollution, environmental regulation should be maintained at a relatively stable level to prevent the inhibition effect of haze pollution pressure on the improvement of regional treatment efficiency. In areas with weaker regulatory intensity, they should make full use of their “innovation offset effect” and, under the intervention of government policies, promote the regional improvement of “three wastes” recycling efficiency and ultimately achieve the goal of efficient regional ecological treatment.

## Figures and Tables

**Table 1:** Descriptive statistics of variables.

Variable	Mean	Median	S.D.	Min	Max
ETE	0.379	0.218	0.382	0.043	1.453
REG	1.455	1.305	0.705	0.450	3.810
HAZ	0.053	0.020	0.059	0.000	0.199
URB	0.548	0.526	0.131	0.340	0.893
ENE	0.101	0.085	0.050	0.038	0.240
IND	0.467	0.487	0.081	0.213	0.577
INO	9.573	9.683	1.484	6.219	12.430
FDI	14.324	14.785	1.540	10.878	16.770

**Table 2:** Threshold significance test.

Threshold	Critical Mass					
	*F*-value	*P*-value	Bootstrap times	1%	5%	10%
Single Threshold	6.257**	0.028	500	9.980	4.646	3.617
Double Threshold	2.261	0.116	500	9.850	4.504	2.682
Triple Threshold	2.739	0.208	500	18.860	8.456	5.961

The *P*-value and the critical mass are obtained by using the “self-sampling method” (bootstrap) with 500 replications. *** *p* < 0.01; ** *p* < 0.05; * *p* < 0.1.

**Table 3:** Results of threshold estimators and confidence intervals.

Model	Threshold Estimators	95% Confidence Intervals
Single Threshold	0.810	[0.600,3.010]
Double Threshold	2.955	[0.600,3.400]
	0.720	[0.600,2.660]
Triple Threshold	0.600	[0.600,2.660]

**Table 4:** Results of dynamic threshold regression.

	Coef.	Std.Err.	z	P > |z|	95% Conf. Interval	
L1.	0.214 ***	0.015	14.01	0.000	0.184	0.244
L2.	0.181 ***	0.013	13.56	0.000	0.155	0.207
URB	0.664 ***	0.095	6.95	0.000	0.477	0.851
ENE	−0.845 ***	0.223	−3.79	0.000	−1.282	−0.408
IND	−0.738 ***	0.092	−7.98	0.000	−0.919	−0.557
FDI	−0.011	0.018	−0.62	0.537	−0.047	0.024
INO	−0.090 ***	0.014	−6.36	0.000	−0.117	−0.062
HAZ(REG ≤ 0.810)	1.168 ***	0.241	4.85	0.000	0.696	1.640
HAZ(REG > 0.810)	−0.105 ***	0.031	-3.39	0.001	−0.166	−0.044
_cons	1.260 ***	0.079	16.01	0.000	1.106	1.415

*** *p* < 0.01; ** *p* < 0.05; * *p* < 0.1.

**Table 5:** Arellano-Bond (AR) (1) and AR (2) tests.

Order	z	Prob > z
AR (1)	−2.34	0.019
AR (2)	1.12	0.264

**Table 6:** Distribution of Chinese provinces with different threshold intervals for each year.

Year	REG ≤ 0.810		REG > 0.810	
	Province	Number	Province	Number
2009	Fujian, Henan, Guangdong, Sichuan, and Guizhou	5	Beijing, Tianjin, Hebei, Shanxi, Neimenggu, Liaoning, Jilin, Heilongjiang, Shanghai, Jiangsu, Zhejiang, Anhui, Jiangxi, Shandong, Hubei, Hunan, Guangxi, Hainan, Chongqing, Yunnan, Shaanxi, Gansu, Qinghai, Ningxia, and Xinjiang	25
2010	Shanghai, Henan, Hunan, Sichuan, and Guizhou	5	Beijing, Tianjin, Hebei, Shanxi, Neimenggu, Liaoning, Jilin, Heilongjiang, Jiangsu, Zhejiang, Anhui, Fujian, Jiangxi, Shandong, Hubei, Guangdong, Guangxi, Hainan, Chongqing, Yunnan, Shaanxi, Gansu, Qinghai, Ningxia, and Xinjiang	25
2011	Shanghai, Zhejiang, Henan, Hunan, Guangdong, and Sichuan	6	Beijing, Tianjin, Hebei, Shanxi, Neimenggu, Liaoning, Jilin, Heilongjiang, Jiangsu, Anhui, Fujian, Jiangxi, Shandong, Hubei, Guangxi, Hainan, Chongqing, Guizhou, Yunnan, Shaanxi, Gansu, Qinghai, Ningxia, and Xinjiang	24
2012	Shanghai, Henan, Guangdong, and Sichuan	4	Beijing, Tianjin, Hebei, Shanxi, Neimenggu, Liaoning, Jilin, Heilongjiang, Jiangsu, Zhejiang, Anhui, Fujian, Jiangxi, Shandong, Hubei, Hunan, Guangxi, Hainan, Chongqing, Guizhou, Yunnan, Shaanxi, Gansu, Qinghai, Ningxia, and Xinjiang	26
2013	Jilin and Guangdong	2	Beijing, Tianjin, Hebei, Shanxi, Neimenggu, Liaoning, Heilongjiang, Shanghai, Jiangsu, Zhejiang, Anhui, Fujian, Jiangxi, Shandong, Henan, Hubei, Hunan, Guangxi, Hainan, Chongqing, Sichuan, Guizhou, Yunnan, Shaanxi, Gansu, Qinghai, Ningxia, and Xinjiang	28
2014	Jilin, Fujian, Hunan, Guangdong, and Hainan	5	Beijing, Tianjin, Hebei, Shanxi, Neimenggu, Liaoning, Heilongjiang, Shanghai, Jiangsu, Zhejiang, Anhui, Jiangxi, Shandong, Henan, Hubei, Guangxi, Chongqing, Sichuan, Guizhou, Yunnan, Shaanxi, Gansu, Qinghai, Ningxia, and Xinjiang	25
2015	Tianjin, Jilin, Henan, Guangdong, Hainan, and Sichuan	6	Beijing, Hebei, Shanxi, Neimenggu, Liaoning, Heilongjiang, Shanghai, Jiangsu, Zhejiang, Anhui, Fujian, Jiangxi, Shandong, Hubei, Hunan, Guangxi, Chongqing, Guizhou, Yunnan, Shaanxi, Gansu, Qinghai, Ningxia, and Xinjiang	24
2016	Tianjin, Liaoning, Jilin, Shanghai, Fujian, Hunan, Guangdong, Hainan, and Chongqing	9	Beijing, Hebei, Shanxi, Neimenggu, Heilongjiang, Jiangsu, Zhejiang, Anhui, Jiangxi, Shandong, Henan, Hubei, Guangxi, Sichuan, Guizhou, Yunnan, Shaanxi, Gansu, Qinghai, Ningxia, and Xinjiang	21

## References

[1] Pan S., Du S., Wang X., Zhang X., Xia L., Jiaping L., Pei F., Wei Y. (2019). Analysis and interpretation of the particulate matter (PM_10_ and PM_2.5_) concentrations at the subway stations in Beijing, China. Sustain. Cities Soc..

[2] Breen M., Seppanen C., Isakov V., Arunachalam S., Breen M., Samet J., Tong H. (2019). Development of TracMyAir Smartphone Application for Modeling Exposures to Ambient PM_2.5_ and Ozone. Int. J. Environ. Res. Public Health.

[3] Yuan F., Huang H. (2018). Image Haze Removal via Reference Retrieval and Scene Prior. IEEE Trans. Image Process..

[4] Rohde R.A., Muller R.A. (2015). Air Pollution in China: Mapping of Concentrations and Sources. PLoS ONE.

[5] Liu H., Fang C., Zhang X., Wang Z., Bao C., Li F. (2017). The effect of natural and anthropogenic factors on haze pollution in Chinese cities: A spatial econometrics approach. J. Clean. Prod..

[6] Hou J., Teo T.S., Zhou F., Lim M.K., Chen H. (2018). Does industrial green transformation successfully facilitate a decrease in carbon intensity in China? An environmental regulation perspective. J. Clean. Prod..

[7] Wang P., Na Xing L., Li F. (2014). Exploration of the Governmental Responsibility in Environmental Pollution Liability Insurance. Adv. Mater. Res..

[8] Sinn H.-W. (2008). Public policies against global warming: A supply side approach. Int. Tax Public Financ..

[9] Zhang M., Li H. (2018). New evolutionary game model of the regional governance of haze pollution in China. Appl. Math. Model..

[10] Hao Y., Peng H., Temulun T., Liu L.-Q., Mao J., Lu Z.-N., Chen H. (2018). How harmful is air pollution to economic development? New evidence from PM_2.5_ concentrations of Chinese cities. J. Clean. Prod..

[11] Li C.K., Luo J.-H., Soderstrom N.S. (2017). Market response to expected regulatory costs related to haze. J. Account. Public Policy.

[12] Shen L., Wang Y. (2018). Supervision mechanism for pollution behavior of Chinese enterprises based on haze governance. J. Clean. Prod..

[13] Yang J., Guo H., Liu B., Shi R., Zhang B., Ye W. (2018). Environmental regulation and the Pollution Haven Hypothesis: Do environmental regulation measures matter?. J. Clean. Prod..

[14] Ma X., Xue T., Waqas A., Wang J. (2019). Study on the impact of regional innovation on the level of carbon pressure under the constraints of environmental regulations. Chin. J. Manag..

[15] Hillman J., Axon S., Morrissey J. (2018). Social enterprise as a potential niche innovation breakout for low carbon transition. Energy Policy.

[16] Yuan S., Guo H. (2018). Bringing the government’s leading role into full play in ecological governance. People Trib..

[17] Guttman D., Young O., Jing Y., Bramble B., Bu M., Chen C., Fürst K., Hu T., Li Y., Logan K. (2018). Environmental governance in China: Interactions between the state and “nonstate actors”. J. Environ. Manag..

[18] Wang S., Xing J., Zhao B., Jang C., Hao J. (2014). Effectiveness of national air pollution control policies on the air quality in metropolitan areas of China. J. Environ. Sci..

[19] Jiang J., Li S., Hu J., Huang J. (2014). A modeling approach to evaluating the impacts of policy-induced land management practices on non-point source pollution: A case study of the Liuxi River watershed, China. Agric. Water Manag..

[20] Jiang X., Hong C., Zheng Y., Zheng B., Guan D., Gouldson A., Zhang Q., He K. (2015). To what extent can China’s near-term air pollution control policy protect air quality and human health? A case study of the Pearl River Delta region. Environ. Res. Lett..

[21] Li X., Qiao Y., Shi L. (2017). The aggregate effect of air pollution regulation on CO_2_ mitigation in China’s manufacturing industry: An econometric analysis. J. Clean. Prod..

[22] Li Y., Zhou S., Jia Z., Ge L., Mei L., Sui X., Wang X., Li B., Wang J., Wu S. (2018). Influence of Industrialization and Environmental Protection on Environmental Pollution: A Case Study of Taihu Lake, China. Int. J. Environ. Res. Public Health.

[23] Qiu L.-Y., He L.-Y. (2017). Are Chinese Green Transport Policies Effective? A New Perspective from Direct Pollution Rebound Effect, and Empirical Evidence from the Road Transport Sector. Sustainability.

[24] Que W., Zhang Y., Liu S., Yang C. (2018). The spatial effect of fiscal decentralization and factor market segmentation on environmental pollution. J. Clean. Prod..

[25] Chen S.M., He L.Y. (2014). Welfare loss of China’s air pollution: How to make personal vehicle transportation policy. China Econ. Rev..

[26] Tang E., Liu F., Zhang J., Yu J. (2014). A model to analyze the environmental policy of resource reallocation and pollution control based on firms’ heterogeneity. Resour. Policy.

[27] Huang S.K., Kuo L., Chou K.-L. (2018). The impacts of government policies on green utilization diffusion and social benefits—A case study of electric motorcycles in Taiwan. Energy Policy.

[28] Chen Y.-H., Wen X.-W., Wang B., Nie P.-Y. (2017). Agricultural pollution and regulation: How to subsidize agriculture?. J. Clean. Prod..

[29] Auffhammer M., Kellogg R. (2011). Clearing the Air? The Effects of Gasoline Content Regulation on Air Quality. Am. Econ. Rev..

[30] Zheng S., Yi H., Li H. (2015). The impacts of provincial energy and environmental policies on air pollution control in China. Renew. Sustain. Energy Rev..

[31] Willis K., Maureaud C., Wilcox C., Hardesty B.D. (2017). How successful are waste abatement campaigns and government policies at reducing plastic waste into the marine environment?. Mar. Policy.

[32] Vagnoni E., Moradi A. (2018). Local government’s contribution to low carbon mobility transitions. J. Clean. Prod..

[33] Zhong M., Li M., Du W. (2015). Can environmental regulation force industrial structure adjustment: An empirical analysis based on provincial panel data. China Popul. Resour. Environ..

[34] Li H., Fang K., Yang W., Wang D., Hong X. (2013). Regional environmental efficiency evaluation in China: Analysis based on the Super-SBM model with undesirable outputs. Math. Comput. Model..

[35] Hou J., Chen H., Xu J. (2017). External Knowledge Sourcing and Green Innovation Growth with Environmental and Energy Regulations: Evidence from Manufacturing in China. Sustainability.

[36] Kuosmanen T.K. (2010). Measurement and analysis of eco-efficiency. An economist’s perspective. J. Ind. Ecol..

[37] Kharel G., Charmondusit K. (2008). Eco-efficiency evaluation of iron rod industry in Nepal. J. Clean. Prod..

[38] Wu J., Li M., Zhu Q., Zhou Z., Liang L. (2018). Energy and environmental efficiency measurement of China’s industrial sectors: A DEA model with non-homogeneous inputs and outputs. Energy Econ..

[39] Kenneth L.R. (2016). Environmental efficiency measurement and the materials balance condition reconsidered. Eur. J. Oper. Res..

[40] Yang L., Wang K.L., Geng J.C. (2018). China’s regional ecological energy efficiency and energy saving and pollution abatement potentials: An empirical analysis using epsilon-based measure model. J. Clean. Prod..

[41] Hansen B.E. (1999). Threshold effects in non-dynamic panels: Estimation, testing, and inference. J. Econ..

[42] Blundell R., Bond S. (1998). Initial conditions and moment restrictions in dynamic panel data models. J. Econ..

[43] Li L., Tang D., Kong Y., Liu D., Yang Y. (2016). A spatial econometric analysis of impact of FDI on urban haze pollution: Case of the Pearl River delta region. Manag. Rev..

[44] Levinson A. (2004). Environmental regulations and manufacturers’ location choices: Evidence from the Census of Manufactures. J. Public Econ..

[45] Stern D.I., Zha D. (2016). Economic growth and particulate pollution concentrations in China. Environ. Econ. Policy Stud..

[46] Zhao D., Sing T.F. (2017). Air pollution, economic spillovers, and urban growth in China. Ann. Reg. Sci..

[47] Wanlley W. (1994). The contribution of environmental regulations to slowdown in productivity growth. J. Environ. Manag..

[48] Yu F., Guo Y., Le-Nguyen K., Barnes S.J., Zhang W. (2016). The impact of government subsidies and enterprises’ R&D investment: A panel data study from renewable energy in China. Energy Policy.

